# Durability of Immunogenicity and Protection of rVSV∆G-ZEBOV-GP Vaccine in a Nonhuman Primate EBOV Challenge Model

**DOI:** 10.3390/v17030342

**Published:** 2025-02-28

**Authors:** Sandra L. Bixler, Amy C. Shurtleff, Melek M. E. Sunay, Kenneth Liu, Ziqiang Chen, Michael Eichberg, Jakub K. Simon, Beth-Ann G. Coller, Sheri Dubey

**Affiliations:** 1 US Army Medical Research Institute of Infectious Diseases, Porter Street, Fort Detrick, MD 21702, USA; sandra.l.bixler.civ@health.mil (S.L.B.); amy.shurtleff@cepi.net (A.C.S.); mmesunay@gmail.com (M.M.E.S.); 2 Merck & Co., Inc., Lincoln Ave., Rahway, NJ 07065, USA; kenneth_liu@merck.com (K.L.); ziqiang.chen@merck.com (Z.C.); michael.eichberg@merck.com (M.E.); jakub.simon@orelconsulting.com (J.K.S.); bethann.coller@gmail.com (B.-A.G.C.)

**Keywords:** Ebola virus, vaccine, nonhuman primates, vesicular stomatitis virus, filovirus

## Abstract

The rVSVΔG-ZEBOV-GP vaccine demonstrated efficacy in preventing Ebola virus (EBOV) disease in a ring vaccination clinical trial conducted during the 2014–2016 West Africa outbreak and is licensed by regulatory agencies, including the US FDA and the EMA. Here, we present two studies that evaluated the durability of immunogenicity and protection from an EBOV challenge up to ~12 months following vaccination with rVSVΔG-ZEBOV-GP in nonhuman primates (NHPs). Cynomolgus macaques were vaccinated with either one or two doses of rVSVΔG-ZEBOV-GP or a saline control and were challenged intramuscularly with EBOV at a target dose of 1000 pfu at ~4 months (Study 1) or ~8 or ~12 months (Study 2) after the last vaccination. All vaccinated animals developed robust ZEBOV-GP-specific IgG and neutralizing antibody titers, which were sustained until the last time point tested prior to the challenge. The majority of animals (88–93%) challenged with EBOV at ~4 or ~8 months post-vaccination survived, whereas the survival rate was lower (53%) in animals challenged ~12 months post-vaccination. These results demonstrate that both one-dose and two-dose regimens of the rVSVΔG-ZEBOV-GP vaccine induced durable ZEBOV-GP-specific antibody titers in NHPs and provided high levels of protection against a lethal EBOV challenge up to ~8 months post-vaccination. In this stringent challenge model, decreased protection was observed at ~12 months post-vaccination despite sustained antibody levels.

## 1. Introduction

Ebola virus (EBOV), formerly known as *Zaire ebolavirus* (ZEBOV), is a filamentous, enveloped ribonucleic acid (RNA) virus classified under the species *Orthoebolavirus zairense* and a member of the *Filoviridae* family [1]. Although the reservoir host for EBOV has not been conclusively identified, bats have been proposed as a likely candidate [2]. Zoonotic transmission of orthoebolaviruses to humans can occur via direct contact with the reservoir host or infected intermediate hosts, as well as through human-to-human transmission [3]. EBOV is often considered the most virulent orthoebolavirus and has been responsible for the majority of Ebola virus disease (EVD) outbreaks, with a case fatality rate that has typically exceeded 70% [4]. In the 2014–2016 EBOV outbreak in West Africa, over 28,000 people were infected, resulting in more than 11,000 deaths [5]. During the 2014–2016 outbreak, there was no approved vaccine or therapeutic available for the prevention or treatment of EVD.

rVSVΔG-ZEBOV-GP is a replication-competent, live, recombinant virus vaccine consisting of the vesicular stomatitis virus (VSV) genetically engineered to express the EBOV glycoprotein (GP) gene (rVSVΔG-ZEBOV-GP) in place of the VSV glycoprotein (G) gene (strain Indiana). The efficacy and safety of rVSVΔG-ZEBOV-GP was demonstrated in a randomized controlled trial conducted during the 2014–2016 outbreak. rVSVΔG-ZEBOV-GP was evaluated in eight Phase 1 [6,7,8,9,10,11,12,13] and five Phase 2/3 [14,15,16,17,18] clinical trials, with more than 18,000 participants vaccinated, and was found to be generally well tolerated [19,20]. Evidence from these trials, including the pivotal Phase 3 “Ebola ça Suffit!” study (PACTR201503001057193) [16], was part of the submissions for licensure to both the US Food and Drug Administration (FDA) and the European Medicines Agency (EMA). The EMA granted conditional (November 2019) and full market authorization (January 2021) for the rVSVΔG-ZEBOV-GP vaccine (ERVEBO^®^, also known as V920; Merck Sharp & Dohme LLC., a subsidiary of Merck & Co., Inc., Rahway, NJ, USA), making it the first vaccine approved for active immunization against EVD in adults aged ≥18 years [21]. The vaccine was subsequently approved for use in adults aged ≥18 by the US FDA in December 2019 [22]. An expanded indication has been approved by the EMA (in September 2023) and the US FDA (in August 2023) to include individuals aged ≥1 year [23,24]. As of December 2023, rVSVΔG-ZEBOV-GP has also been approved in the UK, Canada, Switzerland, and 11 countries in Africa (Burundi, Central African Republic, Cote d’Ivoire, Democratic Republic of the Congo, Ghana, Guinea, The Republic of the Congo, Rwanda, Sierra Leone, Uganda, and Zambia) [25].

Experimental infection of nonhuman primates (NHPs) with EBOV by intramuscular injection or mucosal exposure can recapitulate many of the key features of human EVD [26], making it the preferred model for preclinical studies to evaluate the immunogenicity and efficacy of EBOV vaccines. Previous preclinical studies have shown that a single immunization with the rVSVΔG-ZEBOV-GP vaccine administered at least 28 days prior to challenge is highly immunogenic and protects against lethal disease in the NHP model [27,28,29,30,31,32]. The durability and mechanism of protection of rVSVΔG-ZEBOV-GP against an EBOV challenge in animal models is not currently known, but research is advancing in the characterization of humoral immunity against the virus [33].

Here, we assessed antibody responses and protection after one or two doses of rVSVΔG-ZEBOV-GP in NHPs challenged with EBOV approximately 4 months (Study 1) or approximately 8 or 12 months (Study 2) after the last vaccination. These studies were undertaken to assess the durability of immunogenicity and protection at time points beyond those assessed in published studies where NHPs were challenged at peak immunogenicity (28–42 days post-vaccination). Additionally, these studies were intended to confirm and expand upon preliminary observations in a study of cynomolgus macaques of different geographical origin (Mauritius) vaccinated with a single lower dose of rVSVΔG-EBOV-GP and challenged at 3 or 12 months post-vaccination [33]. The two-dose regimens in the studies described here were administered approximately 2 months apart to align with the two-dose regimen in the PREVAC study [34] and were compared with the single-dose regimen to assess the potential impact of the number of doses on durability. Antibody titers were measured by a validated enzyme-linked immunosorbent assay (ELISA) and a plaque reduction neutralization test (PRNT) in each study cohort over time, and antibody levels were compared in survivors versus non-survivors to assess the durability of the immune response and the potential association of antibody levels with protection.

## 2. Materials and Methods

### 2.1. Study Design

Studies 1 and 2 evaluated the durability of the immunogenicity and efficacy of vaccination with either one or two doses of rVSVΔG-ZEBOV-GP in animals challenged with EBOV approximately 4 months (Study 1) or approximately 8 or 12 months (Study 2) after the last vaccination, where 1 month is defined as approximately 28 days. Both studies were conducted by the United States Army Medical Research Institute of Infectious Diseases (USAMRIID). Study personnel were blinded to which animal belonged to which vaccine group.

In Study 1, 17 cynomolgus macaques (of Cambodian origin) were vaccinated intramuscularly (IM) with either one (Group 1, *n* = 7) or two doses (administered 63 days apart; Group 2, *n* = 8) of rVSVΔG-ZEBOV-GP at the full human clinical dose level of ≥7.2 × 10^7^ plaque-forming units (pfu) or saline (non-vaccinated control; Group 3, *n* = 2). Approximately 4 months (121 days) after the last vaccination, animals were challenged IM with EBOV (strain Kikwit) at a target dose of 1000 pfu. Previous studies have shown this dose of EBOV to be uniformly lethal within 1 week [35]. The titer of the virus inoculum administered to the animals was confirmed by the neutral red plaque assay.

In Study 2, a total of 36 cynomolgus macaques (of Cambodian origin) were vaccinated with either one dose (Groups 2 and 4, *n* = 8 each; Group 5, *n* = 2) or two doses (administered 56 days apart; Groups 1 and 3, *n* = 8 each) of rVSVΔG-ZEBOV-GP at the full human clinical dose level of ≥7.2 × 10^7^ pfu. Approximately 12 months (Group 1, two doses; Group 2, one dose) or 8 months (Group 3, two doses; Group 4, one dose) after the last vaccination, animals were challenged IM with EBOV (strain Kikwit) at a target dose of 1000 pfu. The positive control group (Group 5, *n* = 2) was challenged with EBOV 42 days after vaccination with one dose of rVSVΔG-ZEBOV-GP. The non-vaccinated control group (Group 6, *n* = 2) was administered one dose of saline 42 days prior to the EBOV challenge.

The key endpoints for both studies were survival to Day 28 post-challenge and estimated geometric mean antibody titers (GMTs) prior to each vaccination and after each vaccination.

#### Vaccine and Challenge Virus

A review of the development of the rVSVΔG-ZEBOV-GP vaccine has been documented, and the product has been described previously [20]. In both studies, a clinical-grade rVSVΔG-ZEBOV-GP vaccine candidate manufactured by IDT Biologika was used. The EBOV (strain Kikwit) used for the challenge was isolated from a human case from the Democratic Republic of Congo in 1995 (USAMRIID challenge stock “R4415”) and is the most used EBOV isolate for NHP studies [36,37].

### 2.2. Study Assessments

#### 2.2.1. Humoral Immune Response

Antibody responses to the rVSVΔG-ZEBOV-GP vaccine were measured in serum samples collected during the vaccination phase of each study. In Study 1, serum samples were collected prior to each vaccination and at 7, 14, 28–29, 35, 42–44, 77, and 114 days after each vaccination (pre-challenge). In Study 2, serum samples were collected prior to each vaccination and weekly for the first 2 months after each vaccination, followed by monthly collections until challenge. In both studies, samples were stored frozen until testing at Q^2^ Solutions Vaccines (San Juan Capistrano, CA, USA).

Anti-EBOV GP immunoglobulin G (IgG) titers were determined using the validated human ZEBOV-GP IgG ELISA as previously described [11,38]. The assay was validated for the analysis of both human and NHP samples based on in-depth parallel assessments [39] and was demonstrated to have a lower limit of quantitation of 13.62 ELISA units (EU)/mL at Q^2^ Solutions. The seroresponse for GP-ELISA was assessed in two ways: a 2-fold increase from baseline to 200 EU/mL or higher, or a 4-fold increase from baseline [40]. Virus-neutralizing antibody titers during the vaccination phase were also measured in a validated assay based on a 60% neutralizing titer (PRNT_60_) of the vaccine virus as a Biosafety Level-2 (BSL-2) surrogate for wild-type EBOV, as previously described [11]. This assay was developed by and conducted at Q^2^ Solutions.

#### 2.2.2. Clinical Observations and Responsiveness

During the challenge phase, awake observations were conducted at least once daily and increased in frequency following the development of clinical signs of disease. Animals were assigned a responsiveness score from 0 to 4, with 0 being alert and responsive and 4 being persistently prostrate or severely unresponsive. The frequency of cage-side observations returned to once daily when all surviving animals received a responsiveness score of 0. Anesthetized physical examinations, which included the collection of temperature and weight data, were also performed periodically during the challenge phase.

#### 2.2.3. Survival

Animals that survived until 28 days post-challenge were designated as survivors. Beginning at 28 days post-challenge, survivors were randomized and euthanized on consecutive days (with the exception of holidays or weekends) until all necropsies were completed. The number of days required for necropsy and the number of animals per necropsy were at the discretion of the pathologist.

#### 2.2.4. Blood Viral Levels

EBOV RNA in plasma was measured at USAMRIID for both studies using a validated qRT-PCR method, as previously described [37].

### 2.3. Statistical Analysis

GMTs of the anti-EBOV GP IgG using the validated ZEBOV-rGP ELISA and GMTs of the EBOV GP-specific neutralizing antibody titers to the rVSVΔG-ZEBOV-GP vaccine using the validated rVSVΔG-ZEBOV-GP PRNT_60_ were estimated across all treatment groups on all study days tested. GMT estimates and 95% confidence intervals (CIs) were based on an analysis of variance (ANOVA) model including the treatment group as a categorical covariate. The ANOVA model was applied separately on all study days tested. Other analyses included the estimation of the geometric mean ratios (GMRs) of ZEBOV-rGP ELISA and rVSVΔG-ZEBOV-GP PRNT_60_ between dose groups (one dose vs. two doses [for Study 1 and separately for the 12-month and 8-month challenge groups in Study 2]) and the non-vaccinated control; GMR estimates and 95% CIs (and *p* values for Study 1) were based on the same ANOVA model.

Survival was analyzed for each treatment group using the Kaplan–Meier estimate. Animals alive at the end of in-life assessment were censored at the time of euthanasia. Differences in the resulting survival curves were compared by log-rank tests. The proportion of surviving animals was compared using Fisher’s exact test, and mean time to death (MTTD) was calculated for non-survivors.

The relationship between survival and treatment (one dose vs. two doses [for Study 1 and separately for the 12-month and 8-month challenge groups in Study 2] and the non-vaccinated control [Study 1 only]) or survival and titer response (log[ELISA titer] or log[PRNT titer]) were assessed using univariate Cox proportional hazards analysis models, where the outcome was survival time or censor.

Since these were both estimation studies, no multiplicity adjustments were made. Unless specified otherwise, all statistical testing was two-sided and was performed using a significance (alpha) level of 0.05. All analyses were conducted using the SAS^®^ system Version 9.4 or higher or the R statistical software Version 4.2.1 or higher.

## 3. Results

### 3.1. Antibody Response After Vaccination

**Figure 1:**
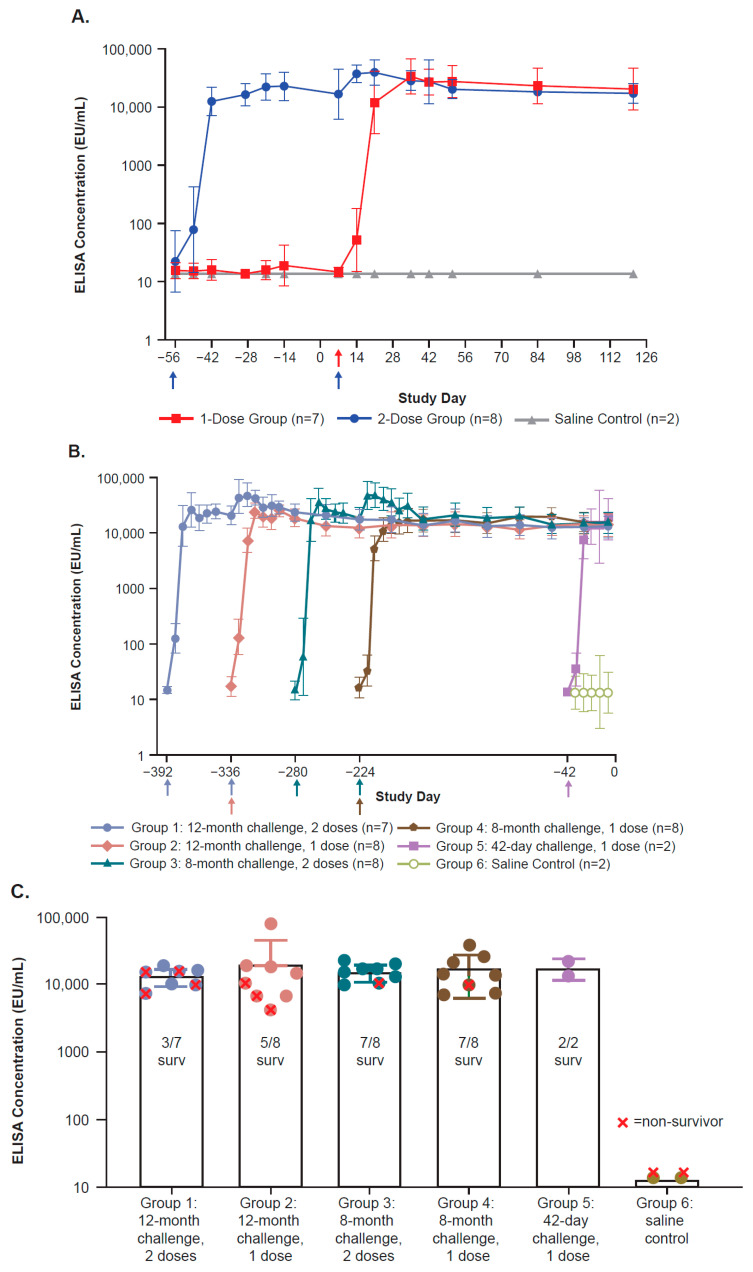
ZEBOV-GP-specific IgG binding antibody responses (geometric mean +/− SD) post-vaccination as measured by ELISA (EU/mL) over time prior to challenge in Study 1 (**A**) and Study 2 ((**B**,**C**) [last sample day prior to challenge]). For samples with ELISA reported as <LLOQ, the LLOQ value of 13.62 EU/mL was used for the calculation of group means. (**A**): Arrows depict the day of rVSVΔG-ZEBOV-GP administration: Day −56 (two-dose group only) and Day 7 (both rVSVΔG-ZEBOV-GP dose groups). ELISA responses from blood collected at the “pre-bleed” study time point are shown at Day −56. Pre-bleed samples were collected prior to Day −56, and no blood samples were collected on Day −56. (**B**): Arrows depict the day of rVSVΔG-ZEBOV-GP administration: Day −392 (Group 1), Day −336 (Groups 1, 2), Day −280 (Group 3), Day −224 (Groups 3, 4), and Day −42 (Group 5 rVSVΔG-ZEBOV-GP, Group 6 saline). (**C**): ELISA concentration (EU/mL) at 7 days before challenge for each group. ELISA, enzyme-linked immunosorbent assay; EU, enzyme units; LLOQ, lower limit of quantitation; mo, month; SD, standard deviation; surv, survivor.

**Figure 2:**
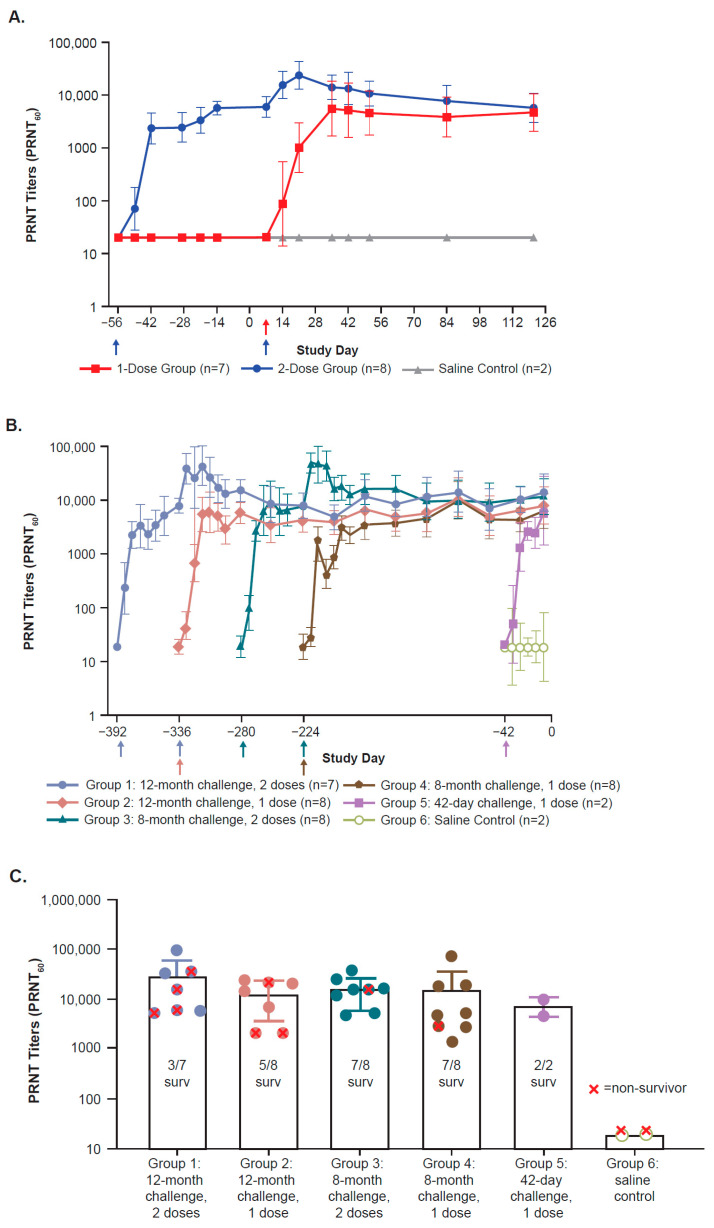
ZEBOV-GP-specific neutralizing antibody responses (mean +/− SD) post-vaccination as measured by PRNT titers (PRNT_60_) over time prior to challenge in Study 1 (**A**) and Study 2 ((**B**,**C**) [last sample day prior to challenge]). The PRNT LLOQ is 35; however, PRNT values were reported down to an LLOD value of 20. For samples with PRNT reported as <20 (less than LLOD), the LLOD value of 20 was used for the calculation of group means. (**A**): Arrows depict the day of rVSVΔG-ZEBOV-GP administration: Day −56 (two-dose group only) and Day 7 (both rVSVΔG-ZEBOV-GP dose groups). PRNT responses from blood collected at the “pre-bleed” study time point are shown at Day −56. Pre-bleed samples were collected prior to Day −56, and no blood samples were collected on Day −56. (**B**): Arrows depict the day of rVSVΔG-ZEBOV-GP administration: Day −392 (Group 1), Day −336 (Groups 1, 2), Day −280 (Group 3), Day −224 (Groups 3, 4), and Day −42 (Group 5 rVSVΔG-ZEBOV-GP, Group 6 saline). (**C**): PRNT titers (PRNT_60_) at 7 days before challenge for each group. LLOD, lower limit of detection; LLOQ, lower limit of quantitation; mo, month; PRNT, plaque reduction neutralization test; SD, standard deviation; surv, survivor.

In Study 1, all vaccinated animals developed robust ZEBOV-GP-specific IgG binding (ELISA; Figure 1A) and neutralizing (PRNT; Figure 2A) antibody titers, which were detectable within 7–14 days after a single vaccination and plateaued by 28–29 days after vaccination. In most animals that received a second dose, ELISA and PRNT titers increased 2- to 8-fold within 14 days; these later reached a plateau similar in magnitude to levels after a single dose. Titers in all animals, regardless of the number of doses, were sustained at these plateau levels through 114 days after the last vaccine dose, which was 7 days prior to the challenge. One animal in the two-dose group had an ELISA response of 437 EU/mL prior to vaccination despite being screened as negative for filovirus prior to study entry. This animal did not have a detectable PRNT titer prior to vaccination, and ELISA levels at the last time point prior to the EBOV challenge were similar to those in other animals in the study.

Similar to Study 1, all vaccinated animals in Study 2 also developed robust ZEBOV-GP-specific IgG binding (ELISA; Figure 1B,C) and neutralizing (PRNT; Figure 2B,C) antibody titers. Antibody responses were detected within 7–14 days after a single vaccination and reached a plateau by 21–28 days post-vaccination. In those animals that received a second dose approximately 2 months after the first dose, ELISA titers increased 2- to 5-fold and PRNT titers increased 5- to 7-fold in most animals within 14 days; these later reached a plateau similar in magnitude to levels after a single dose. Titers in all animals were sustained at these plateau levels and were similar in all vaccination groups 7 days prior to the challenge, which was the last day for which serum samples were available to test prior to moving animals to BSL-4.

### 3.2. Survival Post-EBOV Challenge

**Figure 3:**
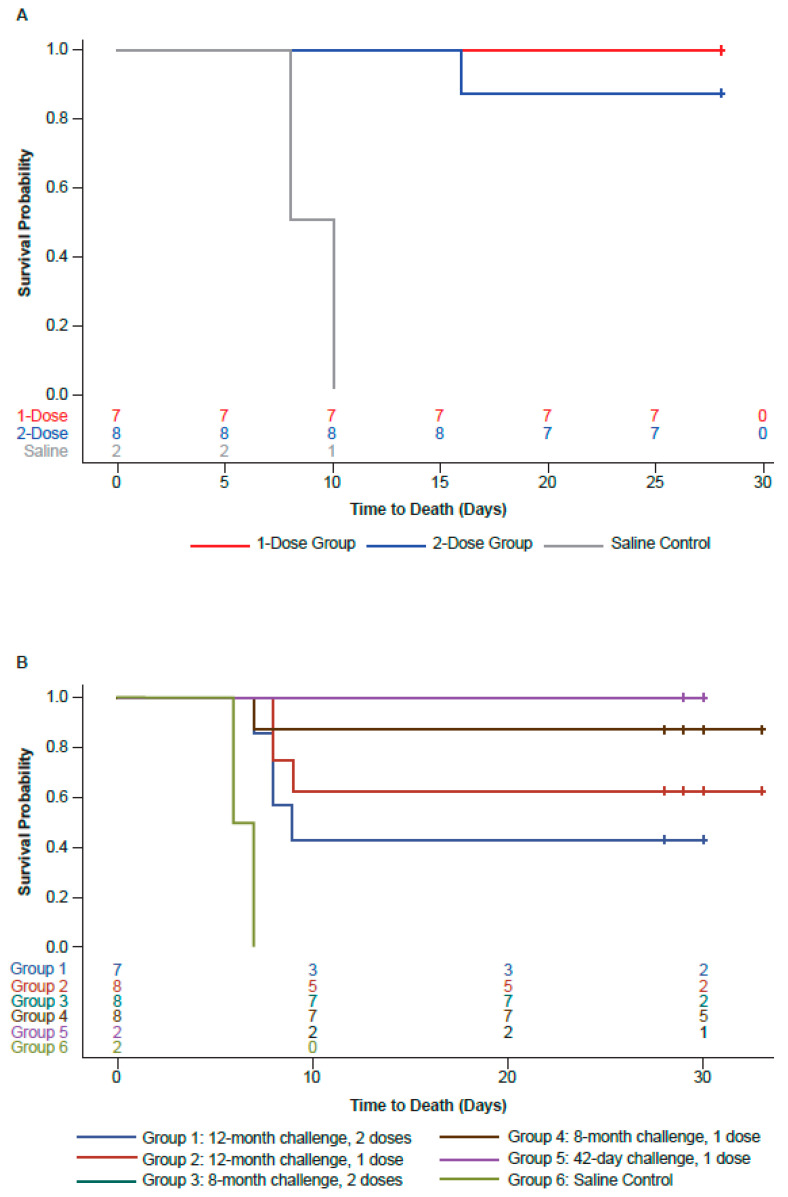
Kaplan–Meier survival plot by vaccination group showing survival outcomes after challenge in (**A**) Study 1 and (**B**) Study 2. Animals alive at the end of in-life assessment were censored at the time of euthanasia (indicated by “+”). Log-rank tests of the survival curve = not significant. Fisher’s test for the percent survival = not significant.

Across both vaccine groups in Study 1, all but one vaccinated animal (93.3%; 14/15) survived following the EBOV challenge (100% [7/7] in the one-dose group and 87.5% [7/8; MTTD = 14 days] in the two-dose group); neither of the non-vaccinated control animals (0/2; MTTD = 14 days) survived following the EBOV challenge (Figure 3A). The sole non-survivor in the two-dose group was euthanized on Day 14 post-challenge due to severe complications arising from inflammation at the site of the EBOV challenge. Log-rank tests of the survival curve, as well as Fisher’s test for the percent survival, showed there was no significant difference between the two vaccine groups.

In Study 2, the survival rate in the animals challenged approximately 12 months after the last vaccination was 63% (5/8; MTTD = 8.33 days) and 43% (3/7; MTTD = 8 days) in the one-dose and two-dose vaccination groups, respectively; the corresponding survival rates in animals challenged approximately 8 months after the last vaccination were 88% (7/8; MTTD = 7 days) and 88% (7/8; MTTD = 14 days), respectively (Figure 3B). Survival was 100% (2/2) in the positive control group challenged with EBOV 42 days after vaccination with one dose of rVSVΔG-ZEBOV-GP and 0% (0/2; MTTD = 6.5 days) in the non-vaccinated control group challenged with EBOV 42 days after the saline dose. Log-rank tests of the survival curve, as well as Fisher’s test for the percent survival, showed that there was no significant difference between the two groups receiving one or two doses of vaccine.

### 3.3. Antibody Titers, Treatment Doses, and Survival Outcomes

To further understand the relationship between antibody responses post-vaccination and survival, we examined the correlation between antibody titers, treatment regimens, and survival outcomes in both studies.

In Study 1, univariate Cox proportional hazards analysis models found that the pre-challenge log(PRNT titer) (Pr > ChiSq = 0.0475) but not the pre-challenge log(ELISA titer) (Pr > ChiSq = 0.2313) at Day 114 after the last vaccination showed an effect on survival following the EBOV challenge. With robust antibody responses in both vaccination groups and so few non-survivors in this study, a threshold level of antibody response predicting survival could not be determined from these data. Additionally, univariate Cox proportional hazards analysis models showed no effect of treatment (one-dose or two-dose vaccination groups), whether the placebo group was excluded (Pr > ChiSq = 0.9984) or included (Pr > ChiSq = 0.9998 and 0.9999), on survival after a challenge with EBOV approximately 4 months after the last vaccine dose (Supplementary Table S1). These results should be interpreted while keeping in mind the small sample sizes and large standard errors.

In Study 2, univariate Cox proportional hazards analysis models showed an effect of the pre-challenge (Day 121) log(ELISA titer) (Pr > ChiSq = 0.0002) and pre-challenge log(PRNT titer) (Pr > ChiSq = 0.0015) on post-challenge survival. A threshold level of antibody response that predicts survival could not be determined from these data. Univariate Cox proportional hazards analysis models that compared one-dose vs. two-dose regimens (excluding placebo) showed no effect of treatment on survival after a challenge with EBOV approximately 12 and 8 months after the last vaccine dose (Pr > ChiSq = 0.4421 and 1.0000, respectively; Supplementary Table S2).

### 3.4. Observations After Challenge

**Figure 4:**
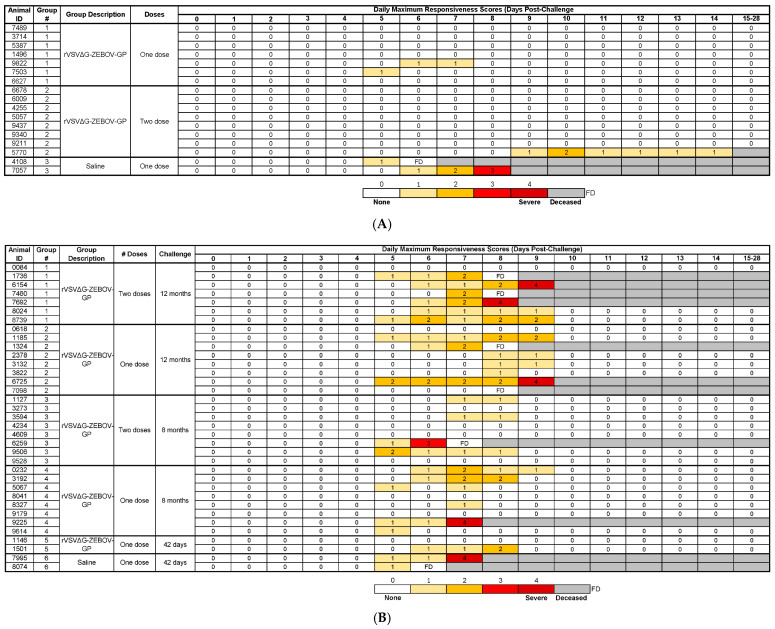
Daily maximum responsiveness scores in Study 1 (**A**) and Study 2 (**B**). Cage-side observations were performed at least once per day, at which time animals were assigned a responsiveness score of 0 to 4. The figure shows scores through Day 15 post-challenge; observations decreased in frequency starting on Day 15 post-challenge, as none of the remaining animals demonstrated responsiveness scores of “1” or greater. FD, found deceased.

Clinical observations, changes in body weight and temperature, clinical pathology findings, and anatomic pathology observations following the EBOV challenge are summarized in Supplementary Table S3. In Study 1, all but one vaccinated animal (in the one-dose group) developed clinical signs post-challenge. Clinical signs in vaccinated animals were nonspecific, including reduced consumption, changes in stool and/or urine output, and inflammation at the challenge site. Only three vaccinated animals (2/7 in the one-dose group and 1/8 in the two-dose group) demonstrated reduced responsiveness and activity, as evidenced by responsiveness scores greater than zero (Figure 4A). The unvaccinated control animals demonstrated classic clinical signs of EVD, such as petechial rash and reduced activity/responsiveness, and rapidly succumbed to EBOV on Days 6–8 post-challenge.

In Study 2, the majority of animals challenged approximately 12 months after receiving one (7/8) or two doses (6/7) of the rVSVΔG-ZEBOV-GP vaccine demonstrated reduced levels of responsiveness and clinical signs such as changes in stool and urine output (Figure 4B). Petechial rash and inflammatory reactions at the challenge site were noted in both survivors and non-survivors. Animals challenged approximately 8 months after the last vaccination with either one dose or two doses developed similar clinical signs to those challenged at approximately 12 months, but these signs were less severe overall. Reduced responsiveness/activity was observed in 6/8 and 4/8 animals in the one-dose and two-dose 8-month post-challenge groups, respectively (Figure 4B). The positive control animals vaccinated 42 days prior to the challenge only displayed mild, transient clinical signs, such as localized rash, facial swelling, and the absence of urine/stool; 1/2 animals had responsiveness scores of greater than 0. Both unvaccinated control animals presented with petechial rash, reduced activity/responsiveness, and inflammation at the challenge site before succumbing to EBOV infection (Supplementary Table S3).

### 3.5. EBOV Viremia After Challenge

**Figure 5:**
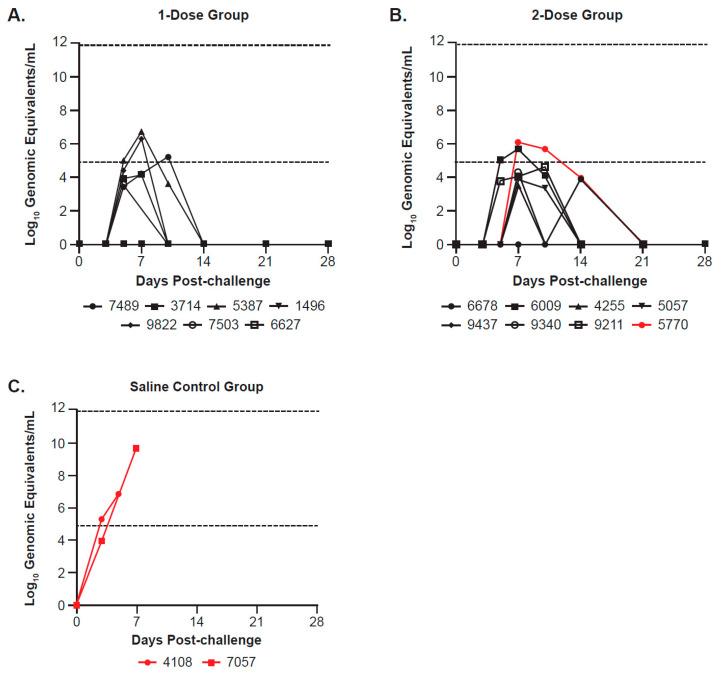
EBOV blood viral levels/plasma viral titers (log10 genomic equivalents/mL) post-challenge among (**A**) the 1-dose, (**B**) 2-dose, and (**C**) saline control groups in Study 1. The figure shows plasma viral loads determined by qRT-PCR for each NHP (individual animal IDs are listed below each graph) at various time points, run in triplicate. Inactivated plasma collected on Days 0, 3, 5, 7, 10, 14, 21, and 28 post-challenge, as well as terminal (where applicable), were evaluated for EBOV RNA by qRT-PCR and reported as log10 genomic equivalents per mL (log10 GE/mL). Symbols and lines for non-survivors in each group are shown in red; the last data point represents the day animals met euthanasia criteria, at which time a terminal sample was collected. The dashed lines at 4.9031 and 11.9031 log10 GE/mL represent the lower limit of quantitation (LLOQ) and upper limit of quantitation, respectively. Values below the LLOQ were transformed for graphing purposes and are reported as LLOQ/√2 (or 3.47 log10 GE/mL). Any samples that were determined to be “undetected” or below the limit of detection were assigned a value of 0 for graphing purposes.

In Study 1, EBOV RNA was either undetected or below the lower limit of quantification in the majority of the vaccinated animals (Figure 5). By Day 7 post-challenge, only 4/15 of the vaccinated animals had a quantifiable viral load: two each in the one-dose and two-dose groups (Figure 5A,B). All vaccinated survivors demonstrated clearance of viral RNA by Day 21 post-challenge. This is in contrast to the two non-vaccinated control animals, which had quantifiable viral loads as early as Day 3 post-challenge and a peak viral load of 6.86 log_10_ genome equivalents (GE)/mL in one of two controls (Figure 5C).

**Figure 6:**
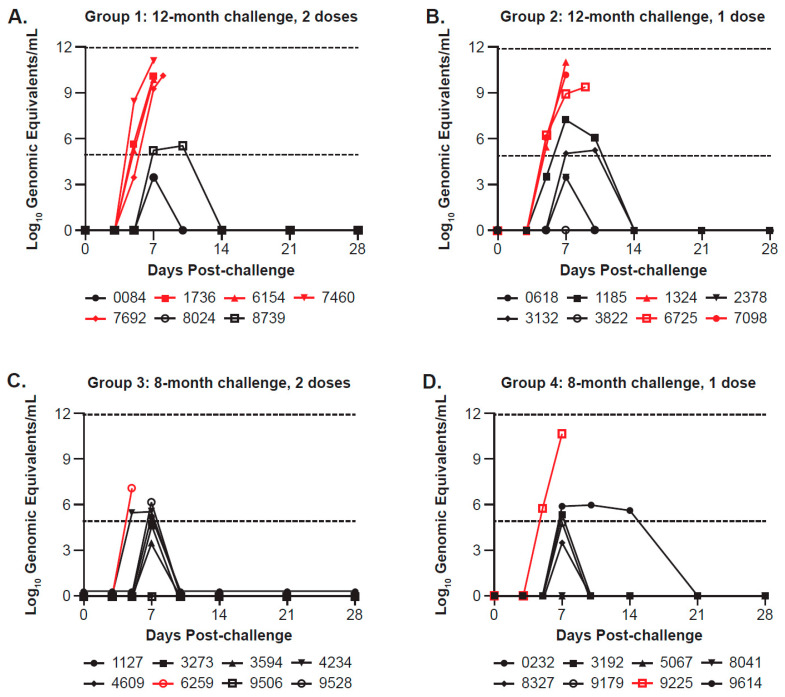

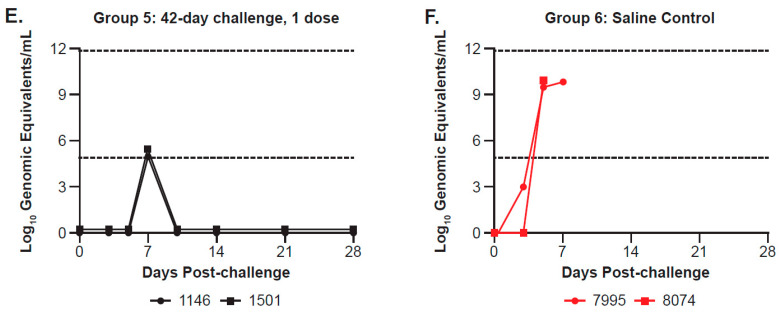
EBOV blood viral levels/plasma viral titers (log10 genomic equivalents/mL) post-challenge among (**A**) Group 1, (**B**) Group 2, (**C**) Group 3, (**D**) Group 4, (**E**) Group 5, and (**F**) Saline Control Group 6 from Study 2. The figure shows plasma viral loads determined by qRT-PCR for each NHP (individual animal IDs are listed below each graph) at various time points, run in triplicate. Inactivated plasma collected on Days 0, 3, 5, 7, 10, 14, 21, and 28 post-challenge, as well as terminal (where applicable), were evaluated for EBOV RNA by qRT-PCR and reported as log10 genomic equivalents per mL (log10 GE/mL). Symbols and lines for non-survivors in each group are shown in red; the last data point represents the day animals met euthanasia criteria, at which time a terminal sample was collected. The dashed lines at 4.9031 and 11.9031 log10 GE/mL represent the lower limit of quantitation (LLOQ) and upper limit of quantitation, respectively. Values below the LLOQ were transformed for graphing purposes and are reported as LLOQ/√2 (or 3.47 log10 GE/mL). Any samples that were determined to be “undetected” or below the limit of detection were assigned a value of 0 for graphing purposes.

In Study 2, 12 vaccinated survivors did not exhibit quantifiable levels of EBOV RNA post-challenge; the proportion of animals with EBOV RNA levels below the limit of quantitation was relatively equal across the one- and two-dose groups challenged at approximately 12 months (2/7 in Group 1, 3/8 in Group 2) or 8 months (3/8 in Group 3 and 4/8 Group 4) after the last vaccination (Figure 6). By Day 7 post-challenge, 3/8 survivors challenged approximately 12 months after the last vaccination and 7/14 survivors challenged approximately 8 months after the last vaccination had quantifiable levels of viral RNA. However, RNA levels in these animals were much lower than levels observed in the control animals and were quickly cleared in most instances. In the two animals challenged 42 days after a single vaccination, the viral load peaked for both animals just above the limit of quantitation on Day 7 before returning to undetectable status (Figure 6E). In the non-vaccinated control group, both animals exhibited high levels of viral RNA (~10 log_10_ GE/mL) by Day 5 post-challenge (Figure 6F). A similar pattern was also noted for vaccinated non-survivors with initial detection on Day 5 post-challenge, reaching similar peak viral RNA levels.

## 4. Discussion

These studies were conducted to evaluate the durability of the immunogenicity and efficacy of either one or two doses of the rVSVΔG-ZEBOV-GP vaccine administered to cynomolgus macaques (of Cambodian origin) that were challenged with EBOV approximately 4 months (Study 1) or approximately 8 or 12 months (Study 2) after the last vaccine dose. These durability studies utilized the full human clinical dose (≥7.2 × 10^7^ pfu/mL). As anticipated, all unvaccinated control animals in both studies succumbed to EBOV infection, with MTTDs of 7 days in Study 1 and 6.5 days in Study 2. In both studies, survival rates were generally high following vaccination with the rVSVΔG-ZEBOV-GP vaccine. In Study 1, all animals in the single-dose group survived, while seven out of eight in the two-dose group survived. In Study 2, animals challenged 12 months post-vaccination showed reduced survival (42.9% and 62.5% in two different groups), while animals challenged 8 months post-vaccination had higher survival rates (87.5%). There was no significant difference in survival between the single- and two-dose groups in either study. Both one-dose and two-dose regimens of the rVSVΔG-ZEBOV-GP vaccine induced robust and durable ZEBOV-GP-specific antibody titers (ZEBOV-GP IgG ELISA and PRNT) in NHP models. Antibody titers were detectable within 7 to 14 days after a single vaccination dose and reached a plateau by 21 to 28 days. In both studies, EBOV RNA was either undetected or below the lower limit of quantitation in the majority of the vaccinated animals, with lower, slightly delayed, and more transient viral loads compared with unvaccinated controls (detectable by Day 3). By Day 7 post-challenge, a small proportion (4/15 in Study 1 and 10/22 in Study 2) of vaccinated animals exhibited quantifiable viral RNA, which was quickly cleared in most instances.

In both studies, both vaccine regimens (one or two doses) were protective against an EBOV challenge approximately 4 months (Study 1) or approximately 8 or 12 months following the last vaccination dose (Study 2). In Study 1, all but one vaccinated animal (14/15: 7/7 in the one-dose group and 7/8 in the two-dose group) survived the EBOV IM challenge 4 months after the last vaccination. Despite antibody titers being similar and robust across the vaccinated groups in Study 2, survival rates dropped from 88% (in both the one- and two-dose groups) in animals challenged approximately 8 months post-vaccination to 63% (one-dose group) and 43% (two-dose group) in animals challenged approximately 12 months post-vaccination. While the vaccine still offered some degree of protection from a challenge at 12 months post-vaccination, the underlying reasons for the decline in efficacy and reduced survival rates of vaccinated NHPs remain unexplained based on these data. Diminished efficacy in these NHP studies in spite of durable antibody titers aligns with our previous study in Mauritian cynomolgus macaques. Despite lower vaccine doses in that study, sustained antibody titers were observed through Month 12, yet protective efficacy diminished in animals challenged at 12 months [33]. This pattern suggests that the immune mechanisms responsible for early post-vaccination protection may differ from those required for sustained protection over time in this stringent NHP model, and the functionality of antibody responses beyond neutralizing capability may contribute to durable protection, as described in the previous NHP study analysis [33]. Early protection shortly after vaccination is critical in an outbreak response, as demonstrated in the ring vaccination trial conducted by the World Health Organization and their partners in Guinea (“Ebola ça Suffit!”), where robust vaccine efficacy was observed within 10 days of receipt of a single dose of the vaccine [16]. The duration of protection conferred by rVSVΔG-ZEBOV-GP in human participants has not been established.

The durable antibody data in these NHP studies align with immune response observations in three Phase 2/3 trials of rVSVΔG-ZEBOV-GP conducted in Liberia (PREVAIL), Guinea (FLW), and Sierra Leone (STRIVE) during the 2013–2016 West African EVD outbreak, where antibody responses peaked at 28 days and persisted through 12 months post-vaccination [41]. Additional long-term follow-up from three Phase 1 trials of rVSVΔG-ZEBOV-GP showed that IgG titers measured by ELISA were sustained for up to 5 years, while neutralizing antibody levels, assessed using a live wild-type EBOV assay, declined over time [42]. This is in contrast to sustained neutralizing titers up to 2 years using the validated PRNT_60_ assay, which measures the neutralization of the vaccine virus as a surrogate for wild-type EBOV [15].

Administration of a second dose of rVSVΔG-ZEBOV-GP was assessed in these studies to evaluate the potential impact on the durability of the response. In animals that received a second dose 2 months later, titers initially increased 2- to 8-fold (across both studies) within 14 days before plateauing at levels similar to those observed in animals that received a single dose. These plateau levels were sustained across all groups until the final pre-challenge measurement, 7 days before the challenges in Study 1 and Study 2. Additionally, no improvement in the survival rate was observed by adding a second dose, suggesting that, while a second dose briefly enhances the humoral response, it does not contribute significantly to long-term protection in this context. The observation in the current study is also consistent with findings from the PREVAC study (NCT02876328), which assessed the immunogenicity and safety of the rVSVΔG-ZEBOV-GP vaccine in children and adults, administered as a single dose or as two doses 56 days apart [34]. In participants who received a second dose, there was a transient boost in antibody concentrations by Month 3, but this boost was not sustained, and 12-month titers were comparable to those observed in the single-dose groups [34]. These results suggest that an alternate timing or strategy for administering the second dose might be required to achieve sustained enhancement of immune memory. Indeed, the PREPARE trial (NCT02788227), which assessed the immunogenicity and safety of the rVSVΔG-ZEBOV-GP vaccine in adults with an occupational risk of exposure to Ebola virus, determined that a second dose of rVSVΔG-ZEBOV-GP given at Month 18 resulted in a several-fold increase in anti-EBOV GP IgG titers that were sustained for at least 18 months [43].

Cox proportional hazards analysis suggested that pre-challenge antibody titers were significant predictors of survival upon a challenge with EBOV, although a specific threshold level of antibody response that predicts survival or time to death could not be determined from these studies. This finding is in line with another analysis of two dose-ranging studies of the rVSVΔG-ZEBOV-GP vaccine in an NHP EBOV challenge model. This analysis determined the impact of the dose level on immune responses and efficacy in an NHP EBOV challenge model, where robust antibody responses were observed at all doses; however, a definitive threshold of protection could not be determined [44]. Subsequent post hoc analysis in that study identified a Day 28 ELISA cutoff for a 50% probability of survival at 978 EU/mL and a PRNT of 132. Despite higher antibody titers in NHPs compared with humans receiving the same or similar doses, efficacy is similarly high in both species. This suggests that titers below these thresholds in NHPs could still be protective. Applying previously reported human ELISA thresholds—at least 200 EU/mL and a 2-fold increase over baseline—has been strongly associated with protection from an EBOV challenge in NHP studies [45] and is consistent with recent post hoc analyses from Phase 2/3 clinical trials of rVSVΔG-ZEBOV-GP suggesting a dichotomous correlate of protection for humans using this seroresponse definition [40]. Furthermore, we have previously shown that the level of antibodies specific for soluble glycoprotein may be a correlate of rVSVΔG-ZEBOV-GP vaccine-mediated protection [33]. This consistency across studies highlights the complexity of EBOV immunity and the need for further investigation into broader immune responses—such as Fc effector function, antibody affinity, and cellular immunity—over time following vaccination to better understand correlates of durable protection.

In summary, vaccination with either one or two doses of the rVSVΔG-ZEBOV-GP vaccine in cynomolgus macaques induced robust and durable ZEBOV-GP-specific antibody titers. Both vaccine regimens were highly protective against IM EBOV challenge at 4 and 8 months post-vaccination, although reduced protection was observed when the animals were challenged 12 months after vaccination. Cox proportional hazards analysis showed that pre-challenge antibody titers were significant predictors of survival upon an EBOV challenge. A specific antibody threshold of antibody response predicting survival could not be determined from these data; however, this result aligns with similar studies suggesting that lower antibody titers may still be protective (an ELISA titer of at least 200 EU/mL or a 2-fold increase over baseline is strongly associated with protection). These findings provide valuable insights into the durability of immune response and protection, which are particularly important in settings where long-term immunity is crucial for outbreak preparedness.

## Data Availability

The data-sharing policy, including restrictions, of Merck Sharp & Dohme LLC., a subsidiary of Merck & Co., Inc., Rahway, NJ, USA (MSD), is available at https://trialstransparency.msdclinicaltrials.com/policies-perspectives.aspx. Requests for access to the clinical study data can be submitted via email to the Data Access mailbox (dataaccess@msd.com).

## Supplementary Materials

The following supporting information can be downloaded at https://www.mdpi.com/article/10.3390/v17030342/s1, Table S1: Cox proportional hazards survival analysis by vaccination group in Study 1; Table S2: Cox proportional hazards survival analysis by vaccination group in Study 2; Table S3: Observations after challenge.
