# Supplementary material for: Durability of Immunogenicity and Protection of rVSV∆G-ZEBOV-GP Vaccine in a Nonhuman Primate EBOV Challenge Model
Source: Viruses. 2025 Feb 28;17(3):342. doi: 10.3390/v17030342 (PMC11945958; doi:10.3390/v17030342)
Supplement: Supplementary file 1 [file viruses-17-00342-s001.zip › viruses-3457159-supplementary.pdf]

Durability of Immunogenicity and Protection of rVSVΔG-ZEBOV-GP Vaccine in a Nonhuman Primate EBOV Challenge Model. Bixler et al.

SUPPLEMENTARY MATERIALS

SUPPLEMENTARY TABLES

Supplementary Table 1. Cox proportional hazards survival analysis by vaccination group in Study 1

Parameter	Degrees of Freedom	Parameter Estimate	Standard Error	Chi-Square	Pr > ChiSq	Hazard Ratio
Excluding Placebo						
1-Dose	1	-18.07626	8999	0.0000	0.9984	0.000
Including Placebo						
1-Dose	1	-43.56037	147472	0.0000	0.9998	0.000
2-Dose	1	-26.28602	147349	0.0000	0.9999	0.000

Supplementary Table 2. Cox proportional hazards survival analysis by vaccination group in Study 2

Parameter	Degrees of Freedom	Parameter Estimate	Standard Error	Chi-Square	Pr > ChiSq	Hazard Ratio
8-month Challenge Group Time-To-Event = 1-dose vs 2-dose (Excluding Placebo)						
1-Dose	1	0	1.41421	0.0000	1.0000	1.000
12-month Challenge Group Time-To-Event = 1-dose vs 2-dose (Excluding Placebo)						
1-Dose	1	0.58753	0.76443	0.05907	0.4421	0.556

Supplementary Table 3. Observations after challenge

**Clinical Observations**	**Study 1**	One animal in Group 1 and one animal in Group 2 showed no signs of disease. Groups 1 and 2 exhibited mostly nonspecific signs, with reaction at challenge site, changes in urine/stool, and reduced consumption being the most common. Two animals in Group 2 had testicular edema. Group 3 controls developed typical EVD symptoms, including rash and reduced responsiveness, and died rapidly.
	**Study 2**	The most common clinical signs in Groups 1 through 4 were changes in urine/stool, reaction at the challenge site, and/or rash, which were present in both survivors and non-survivors. Group 5 had similar clinical signs to Groups 1–4, although these were mild. Both animals in Group 6 had reactions at the challenge site and rash prior to succumbing to EBOV infection.
**Body Weight**	**Study 1**	Only mild fluctuations in body weight were observed across all groups.
	**Study 2**	Minor fluctuations in body weight were observed across all groups.
**Body Temperature**	**Study 1**	In Group 1, only one animal had an elevated temperature on Day 7 post-challenge. The Group 2 non-survivor also had an elevated temperature on Day 7 post-challenge. Animals in Group 3 did not have elevated temperatures, possibly due to rapid disease progression or infrequent physical exams.
	**Study 2**	A subset of animals in Groups 1 through 4 demonstrated elevated temperatures, regardless of survival status. One animal in Group 1 demonstrated hypothermia prior to death. Group 5 showed no fever. In Group 6, one animal had fever, while another exhibited hypothermia due to end-stage disease.
**Clinical Pathology: Hematology**	**Study 1**	Group 1 had early changes in immune cell populations and platelet alterations following challenge; Group 2 showed similar changes but slightly delayed. Group 3 exhibited lymphopenia and severe changes in RBC parameters consistent with EBOV infection.
	**Study 2**	Regardless of group, both survivors and non-survivors exhibited evidence of RBC turnover, decreased platelets, increased WBC counts, and increased neutrophils, with changes more pronounced and persistent in non-survivors.
**Clinical Chemistry/Serum Chemistry**	**Study 1**	Group 1 showed mild to moderate increases in liver enzymes and CK, as well as moderate to marked increases in CRP. Group 2 had similar alterations to Group 1, with the most pronounced changes in the non-survivor. Group 3 exhibited severe changes indicating hepatocellular damage, kidney dysfunction, and GI inflammation and pathology.
	**Study 2**	Across Groups 1 through 5, survivors had increased ALT, AST, and/or ALP levels suggesting hepatocellular damage and inflammation, as well as increased CK and CRP levels potentially in response to infection and/or inflammation at the challenge site. Non-survivors had similar changes, but they were more pronounced. Non-survivors also had increased BUN and creatinine indicative of renal dysfunction as well as decreased albumin and electrolytes, indicative of inflammatory responses and potential renal or GI dysfunction.
**Coagulation**	**Study 1**	Group 1 and Group 2 only showed minor changes in AT and fibrinogen, likely related to inflammation. Group 3 showed severe coagulation abnormalities consistent with DIC.
	**Study 2**	In survivors across Groups 1 through 5, there were no significant changes in prothrombin time, thrombin time, anti-thrombin, or d-dimers; however, some survivors had elevated aPTT and fibrinogen levels. Regardless of group, non-survivors had increased aPTT, PT, TT, fibrinogen, and d-dimers, and decreased AT, indicating coagulopathy and DIC.
**Anatomic Pathology: Necropsy and Gross Observations**	**Study 1**	Group 1 had minimal findings. Most common findings in Group 2 were inflammation and necrosis at the challenge site. Lung discoloration was present in both Group 1 and Group 2, likely a euthanasia artifact. Group 3 had extensive pathology in multiple tissues including liver, kidney, and spleen, consistent with EBOV infection.
	**Study 2**	Group 5 survivors had no gross necropsy findings. A small subset of survivors in Groups 1, 2, and 3 had enlarged lymph nodes and stomach discoloration. Group 4 had more gross pathology in the liver, lymph nodes, abdominal cavity, and challenge site. All non-survivors, regardless of group, had pathologic abnormalities consistent with EVD, such as macular rash and enlargement and/or discoloration of key organs.

aPTT, activated partial thromboplastin time; AT, Antithrombin; BUN, blood urea nitrogen; CK, creatinine kinase; CRP, C-reactive protein; DIC, disseminated intravascular coagulation; EBOV, Ebola virus; EVD, Ebola virus disease; GI, gastrointestinal; PT, prothrombin time; RBC, red blood cell; TT, thrombin time; WBC, white blood cell.
